# Exponentially weighted moving average—Moving average charts for monitoring the process mean

**DOI:** 10.1371/journal.pone.0228208

**Published:** 2020-02-14

**Authors:** Saowanit Sukparungsee, Yupaporn Areepong, Rattikarn Taboran

**Affiliations:** Department of Applied Statistics, Faculty of Applied Science, King Mongkut’s University of Technology North Bangkok, Bangkok, Thailand; University of Glasgow, UNITED KINGDOM

## Abstract

This research aimed to propose a newly-mixed control chart called the Exponentially Weighted Moving Average—Moving Average Chart (EWMA-MA) to detect the mean change in a process underlying symmetric and asymmetric distributions. The performance of the proposed control chart are compared with Shewhart, MA, EWMA, MA-EWMA and EWMA-MA control charts by using average run length (ARL), standard deviation of run length (SDRL), and median run length (MRL) as the criteria for measuring efficiency which evaluated by using Monte Carlo simulation (MC), Moreover, the proposed control chart will be applied to real data. The results of performance comparison showed that the presented control charts performed better detection than the Shewhart, MA, and EWMA charts. However, the results of detection tended to be slower than those for the MA-EWMA chart. The value of ARL_1_ for the mixed control chart depends on the parameters of the statistics for such control chart. The EWMA-MA chart is a variable following *λ* and the MA-EWMA chart is varied according to *w*. From applying the proposed control chart to the data for flow in the Nile River and data of the real GDP growth (%) in the Lebanese economy, it was found to be in accordance with the research results.

## Introduction

Control charts are a fundamental tool of SPC, control charts are now widely used, not only in industry, but also in many other areas with real applications, such as health care [1], manufacturing processes [2], environmental sciences [3], etc. Shewhart [4] developed the first control chart considered as the main tool of SPC using statistical principles in generating. It is sometimes called the Shewhart chart, which is a chart using the data of the previous production process to scatter a plot and consider the production process. Thus, the pattern of the scatter plot cannot be seen if the production process does not change significantly. For this reason, the Shewhart chart is good at detecting larger shifts in the process. Later, Page [5] and Roberts [6] invented a control chart that could detect changes in the production process, even if the change was only slight, called the cumulative sum (CUSUM) chart and the exponentially weighted moving average (EWMA) chart, which makes them more sensitive than Shewhart chart for detecting the smaller and moderate shifts in the process [7]. In 2004, Khoo [8] studied the MA control chart for detecting the fraction of non-conforming observations and showed that the MA chart had better efficiency than the p chart. Many authors focus on the designing of EWMA and MA charts for various situations. Some researchers, such as Sukparungsee and Areepong [9], proposed an algorithm to design the EWMA chart for detecting the process mean in case of normal distribution. The algorithm was generated from combining martingale and integral equations for finding optimal designs of EWMA procedure. Areepong and Sukparungsee [10] studied the ARL was estimated using the integral equation for the EWMA control chart. When the data is distributed as lognormal, the results are compared by using simulation techniques, and it was found that the integral equations were more efficient than the MC. Khan et al. [11] proposed a EWMA control chart for exponential distributed quality based on moving average statistics, the proposed control chart first transforms the sample data to approximate normal variables, then calculates the moving average (MA) statistic for each subgroup, and finally constructs the EWMA statistic based on the current and the previous MA statistics. It has been observed that the proposed control chart is more efficient in the detection of process for all shift parameters. Aslam et al. [12] proposed a new mixed acceptance sampling plan based on the exponentially weighted moving average (EWMA) statistic. The results showed that the proposed plan is much better than existing one in minimizing the inspection cost and the application of the proposed plan in the industry is given, the proposed plan can be used in the industries for the inspection of the product. Alghamdi et al. [13] studied moving average chart for the Weibull distribution under the time truncated life test by assuming that the failure time of a product follows the Weibull distribution. It was found that the proposed control chart is more efficient in detecting a shift in the process as compared with the existing time truncated control chart. Aslam et al. [14] presented the designing of the X-bar control chart under the symmetry property of normal distribution using the neutrosophic exponentially weighted moving average statistics and evaluated the neutrosophic average run length using the neutrosophic Monte Carlo simulation as the criteria for measuring efficiency. The research results reveal that the theoretical comparisons in the NARL and simulation study showed that the proposed chart had higher efficiency in detection of the change. Moreover, Aslam et al. [15] studied a control chart using normal transformation and generally weighted moving average (GWMA) statistic when the quality characteristic follows the exponential distribution. The results indicated that the proposed control chart had higher efficiency in quick detection of the out-of-control process and quick response of the out-of-control process has been examined on different levels of process shifts for different combinations of the proposed chart parameters. To improve the efficiency of control charts some mixed type control charts have been considered. Wong et al. [16] developed the simple design method and introduced approaches in the steps for the MA chart and the plan of combination for MA-Shewhart chart with a simple technique used with the engineering process. Aslam et al. [17] proposed the np-HEWMA chart and np-EWMA chart for process monitoring when the quality characteristic of interest follows a normal distribution. the proposed np-HEWMA chart has this ability to detect a small shift in the manufacturing process. In 2012, Abbas et al. [18] proposed the EWMA–CUSUM charts for monitoring correlated data using the Average Run Length, extra quadratic loss, and relative Average Run Length as criteria to measure the efficiency with Shewhart, CUSUM, EWMA, Shewhart-CUSUM, and Shewhart-EWMA charts. The newly proposed control charts have efficiency in detecting better than the compared charts. In 2014, Zaman et al. [19] proposed the CUSUM‐EWMA chart to detect the change of variation in the process using the ARL, extra quadratic loss, and relative Average Run Length as criteria to measure the efficiency with Shewhart, EWMA and CUSUM charts. It was found that the CUSUM‐EWMA chart had better efficiency for detection than the control charts of Shewhart, CUSUM-S^2^, S^2^-EWMA, CS-EWMA, floating T-S^2^, floating U-S^2^, classical EWMA, and CUSUM charts. Many authors designed MEC and MCE control charts for various situations including, for example, Aslam [20], Zaman et al. [21], Lu [22], Osei-Aning et al. [23] and Riaz et al [24]. Recently, Aslam et al. [25] proposed the DMA-EWMA chart with the data of exponential distribution. The results found that the proposed chart has the efficiency in detecting changes better than the proposed chart in the research of Khoo and Wang [26].

Therefore, the researcher decided to study for proposed a new control chart by combining the EWMA chart with the MA chart, called the EWMA-MA chart, which is used for detecting the mean changes of the process by comparing the efficiency of the EWMA-MA chart with the MA-EWMA, Shewhart, EWMA, and MA charts. If any chart gives the lowest ARL, it means such a chart has the best efficiency to detect changes. Moreover, this can also be applied to the real data for flow in the Nile River and data of the real GDP growth (%) in the Lebanese economy, which is a normal distribution.

## Moving Average (MA), Exponentially Weighted Moving Average (EWMA), mixed EWMA-MA and performance measures evaluation

The control chart was first used in 1924. The first person who initiated and applied the control chart for controlling the production process was Dr. Walter Andrew Shewhart. The control chart performs three main functions. The first function is to define the production standards and the second function is to facilitate the production process to achieve the goal. The last function is to improve the production process. A control chart could be classified into 2 types: variables and attribute control charts. A variable chart is used in controlling the production process and has measurable features of attributes, i.e., $\bar{X}$ chart, R chart, and S charts. An attribute chart is used for controlling the production process with the measurement of product quality by counting, including the p chart, c chart, np chart, etc. In this research, the related control charts are as follows:

### Moving Average chart (MA chart)

For the MA chart [8], the mean is found following each *w* of time. Assuming that we have *k* random samples of size *n*≥1, and suppose that ${\bar{X}}_{1},\ldots ,{\bar{X}}_{k}$ are independent and identically distributed (i.i.d.) in the time domain are the average of the *i*^th^-sample for *i* =1,…,*k*, the value can be found as follows:

$${\bar{X}}_{i}=\frac{X_{i1}+X_{i2}+X_{i3}+\ldots +X_{in}}{n}.$$

At time *i*, the statistics of the *MA*_*i*_ control chart are calculated from finding the mean of each *w* time by calculating the means of the sub-sample ${\bar{X}}_{i},{\bar{X}}_{i-1},\ldots$ which can be divided into two cases as follows:

$$MA_{i}=\left\{ \begin{array}{l} \frac{{\bar{X}}_{i}+{\bar{X}}_{i-1}+{\bar{X}}_{i-2}+\ldots }{i}\;,i<w\\ \frac{{\bar{X}}_{i}+{\bar{X}}_{i-1}+\ldots +{\bar{X}}_{i-w+1}}{w}\;,i\geq w \end{array}\right.$$ (1)

where *w* is the width of the MA control chart, the mean and variance of statistics *MA*_*i*_ are:

$$E\left(MA_{i}\right)=E\left({\bar{X}}_{i}\right)=\mu$$

$$Var\left(MA_{i}\right)=\left\{ \begin{array}{l} \frac{\sigma^{2}}{ni}\;,\;i<w\\ \frac{\sigma^{2}}{nw}\;,\;i\geq w. \end{array}\right.$$ (2)

Therefore, the control limits of the MA control chart are following

$$UCL/LCL=\left\{ \begin{array}{l} \mu \pm \frac{H_{1}\sigma }{\sqrt{ni}}\;,\;i<w\\ \mu \pm \frac{H_{1}\sigma }{\sqrt{nw}}\;,\;i\geq w \end{array}\right.$$ (3)

where *H*_*1*_ is a coefficient of control limit of MA control chart, *μ* is the mean and *σ*^2^ is the variance of the process under control.

### Exponentially Weighted Moving Average chart (EWMA chart)

The EWMA control chart was introduced by Roberts [6] (see also Lucas and Saccucci [27]), which is suited to detect a small change in process parameters. An EWMA control chart for monitoring the mean of a process is based on the statistic.

$$Z_{i}=\lambda {\bar{X}}_{i}+\left(1‑\lambda \right)Z_{i-1}\;,i=1,2,\ldots$$ (4)

where *λ* is the weighing parameter of the data in the past having the values from 0 to 1, and ${\bar{X}}_{i}$ is the mean of the process at time i. At the very first time point *Z*_0_ = *μ*_0._ (the steady and initial value), where *X*_*i*_ (*i* = 1,2,…) are independent and normally distributed observations, the statistic *Z*_*i*_ for sampling means, ${\bar{X}}_{i}$ should be used, instead of *X*_*i*_ in Eq (4), and $\sigma_{\bar{X}}=\sigma /\sqrt{n}$ should be used instead of *σ* in Eqs (5) and (7), then the mean and variance of *Z*_*i*_ are:

$$E\left(Z_{i}\right)=\mu_{0}$$

$$Var\left(Z_{i}\right)=\sigma_{\bar{X}}^{2}\left(\frac{\lambda }{2‑\lambda }\left(1‑{\left(1‑\lambda \right)}^{2i}\right)\right)\;;i=1,2,\ldots$$ (5)

From Eq (5), when *i*→∞, then the asymptotic variance is

$$Var\left(Z_{i}\right)=\sigma_{\bar{X}}^{2}\left(\frac{\lambda }{2-\lambda }\right).$$ (6)

Therefore, the control limits of the EWMA control chart are following

$$UCL/LCL=\mu_{0}\pm H_{2}\sqrt{\sigma_{\bar{X}}^{2}\left(\frac{\lambda }{2‑\lambda }\right)}$$ (7)

where *H*_*2*_ is a coefficient of control limit of EWMA control chart, *μ*_0_ is the mean of the process and variance is $\sigma_{\bar{X}}^{2}.$

### Mixed Moving Average—Exponentially Weighted Moving Average Chart (MA-EWMA chart)

The MA-EWMA chart was presented by Taboran et al. [28]. The chart was the combination of MA and EWMA control chart. In the mathematical model developed for the MA-EWMA chart design, the plot statistic *Z*_*i*_ of the EWMA chart is used as an input to the MA chart (Eq (1)). Therefore, the statistic of MA-EWMA chart as follows:

$$MA_{i}=\left\{ \begin{array}{l} \frac{Z_{i}+Z_{i-1}+Z_{i-2}+\ldots }{i}\;,\;i<w\\ \frac{Z_{i}+Z_{i-1}+\ldots +Z_{i-w+1}}{w}\;,\;i\geq w. \end{array}\right.$$ (8)

Thus, the asymptotical control limit of MA-EWMA control chart is as follows:

$$UCL/LCL=\left\{ \begin{array}{l} \mu_{Z}\pm H_{3}\sqrt{\left(\frac{\sigma_{Z}^{2}}{i}\right)\left(\frac{\lambda }{2-\lambda }\right)}\;,\;i<w\\ \mu_{Z}\pm H_{3}\sqrt{\left(\frac{\sigma_{Z}^{2}}{w}\right)\left(\frac{\lambda }{2-\lambda }\right)}\;,\;i\geq w \end{array}\right.$$ (9)

where *H*_*3*_ is a coefficient of control limits of MA-EWMA control chart, *μ*_*Z*_ is the mean of the process and variance is $\sigma_{Z}^{2}.$

### Mixed Exponentially Weighted Moving Average—Moving Average Chart (EWMA-MA chart)

The EWMA-MA chart was generated from combining the EWMA chart with the MA chart. Those charts were effective alternatives to EWMA and MA charts. The statistics still belong to the EWMA chart, as shown in Eq (4).

$$Z_{i}=\lambda MA_{i}+\left(1‑\lambda \right)Z_{i‑1}\;,i=1,2,\ldots$$ (10)

where *λ* is the weighing parameter of the data in the past having the values from 0 to 1, *Z*_0_ is the starting value and is set to be equal to the target mean *μ*_0_, then the UCL and LCL of the EWMA-MA chart are the expected values for the data, which will be the same value of the MA chart. Variance will be applied between the EWMA and MA charts, as shown in Eq (2) and Eq (6). The control limits are as follows:

$$UCL/LCL=\mu_{MA}\pm H_{4}\sqrt{\left(\frac{\sigma_{MA}^{2}}{w}\right)\left(\frac{\lambda }{2‑\lambda }\right)}$$ (11)

where *H*_*4*_ is the coefficient of the control limits for the EWMA-MA chart, *μ*_*MA*_ is the mean of the process and variance is $\sigma_{MA}^{2}.$

## Performance measurement methodology

There are several methods for measuring the efficiency of control charts. The most popular measures of the performance are average run length (ARL). ARL is the sample of points under the control limit prior to the signaling process to access the control limit for the first time. ARL is considered in 2 cases. ARL_0_ is used in considering the in-control process, while ARL_1_ is used in considering the out-control process. The mean of the RL distribution is the ARL, the standard deviation of the run length (SDRL) is also computed. The control charts having the best efficiency will give the least ARL_1_ of the control charts. That means such a control chart can detect changes in the mean of the process the soonest. However, the disadvantage of the ARL is the skewness of the run length distribution changes from highly skewed when the process is in-control to approximately symmetric when the process mean shift is large, interpretation based on ARL alone could be erroneous [29]. Therefore, the MRL are used as the criteria for measuring the efficiency of the non-normality cases, which is more credible since it is less affected by the skewness of the run length distribution [30, 31].

In this research, the ARL, SDRL, and MRL are used as the criteria for measuring the efficiency of the control charts which evaluated by using Monte Carlo simulation (MC), MC is simple to program and is adapted for controlling and testing accuracy, which are the estimation of ARL, SDRL, and MRL generated from the creation of the program simulated for finding ARL, SDRL, and MRL which can be found as follows:

$$ARL=\frac{\sum_{t=1}^{N}RL_{t}}{N}$$

$$SDRL=\sqrt{E{\left(RL\right)}^{2}-ARL^{2}}$$

**Fig 1:**
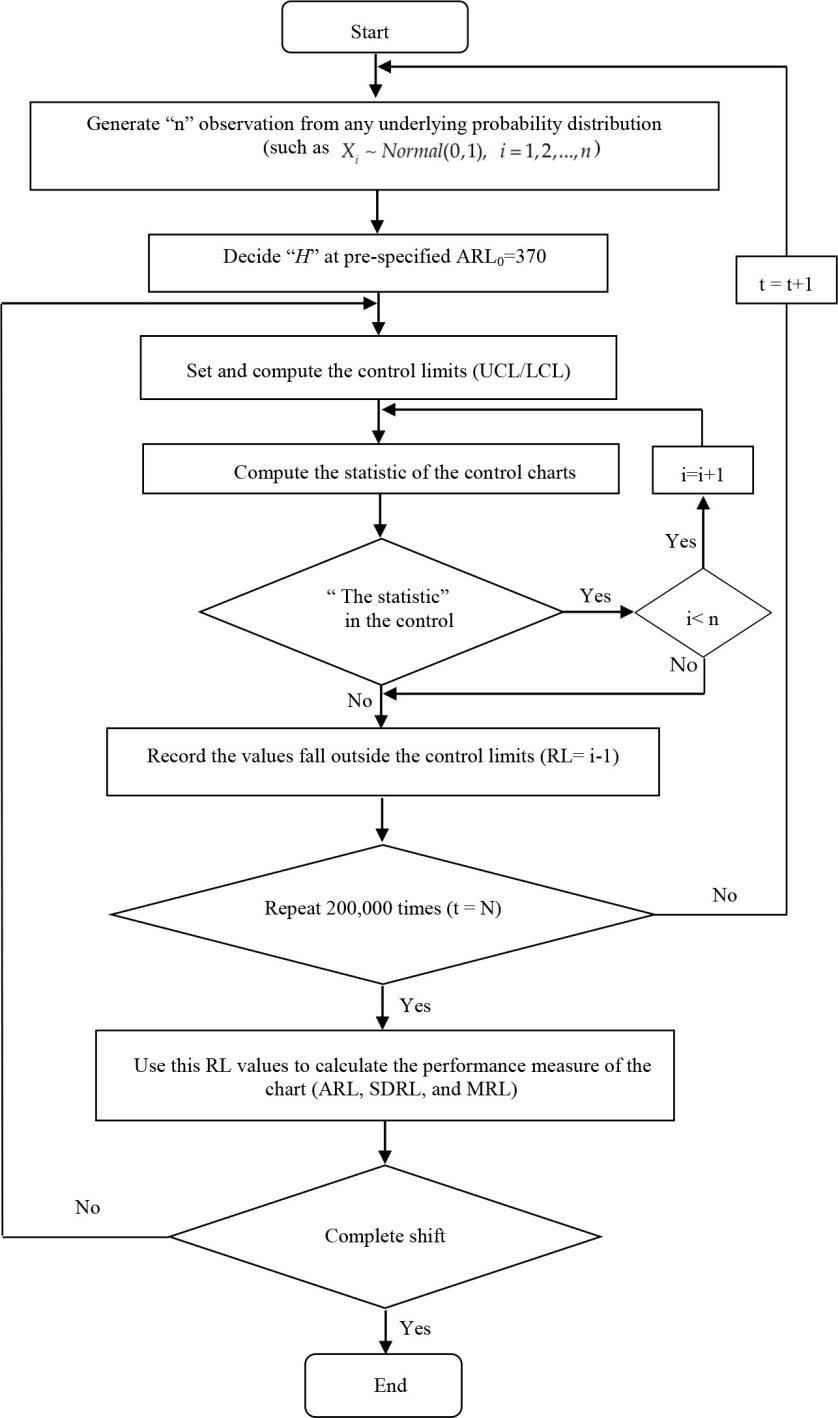
This is the Fig 1 Procedural flow of finding ARL, SDRL, and MRL with MC for control charts.

MRL=Median(RL) (12)

where *RL*_*t*_ is the number of samples before the out-control process being detected for the first time in simulating the data of *t*, while N is the number of repetitions in the simulation, and the values are set as follows: 1) Set the sample size (n) of each round of experiment at 10,000, 2) Set the number of the experiment repetition (N) at 200,000, and 3) Set ARL_0_ = 370 when the process is under control. In this research, the Monte Carlo Simulation (MC) is applied for the EWMA, MA-EWMA, and EWMA-MA charts, as shown in Fig 1.

## Evaluation methods

This research studied the performance of the proposed control chart compared with the Shewhart, MA, EWMA, and MA-EWMA charts when the process is not under control. The study was conducted with 6 distribution processes divided into symmetric distributions, which are Normal(0,1), Laplace(0,1), Logistic(6,2), and Student *t*_10_ distributions and asymmetric distributions with skew to the right, which are Exponential(1) and Gamma(4,1). The determination of the moving average period (*w*) of the MA chart equal to 5. When the data has Normal(0,1), Laplace (0, 1), Logistic(6,2), Student *t*_10_, Gamma(4,1) and Exponential (1) distribution, we have used location shifts in this format: *μ*_1_ = *μ*_0_+*δσ*_0_ where *δ* refers to the amount of shift, *μ*_1_ is the shifted mean, *μ*_0_ is the in-control mean and, *σ*_0_ is the controlled value of process standard deviation. The parameters for each chart were defined such that the ARL when the process is under control was equal to 370. Set the sample size of each round of experiment at 10,000 and the number of experiment repeat above process for the Monte Carlo simulation (MC) at 200,000 cycles for finding the ARL, SDRL, and MRL. The programs used to process the results are R program.

## Research results

According to the comparison of efficiency for the Shewhart, MA, EWMA, EWMA-MA, and MA-EWMA charts at various levels of changes by considering ARL, SDRL, and MRL when the in control processes are from Normal(0,1), Laplace (0, 1), Logistic(6,2), Student *t*_10_, Gamma(4,1) and Exponential (1) distributions with details as follows:

### Proposed control chart (EWMA-MA)

**Fig 2:**
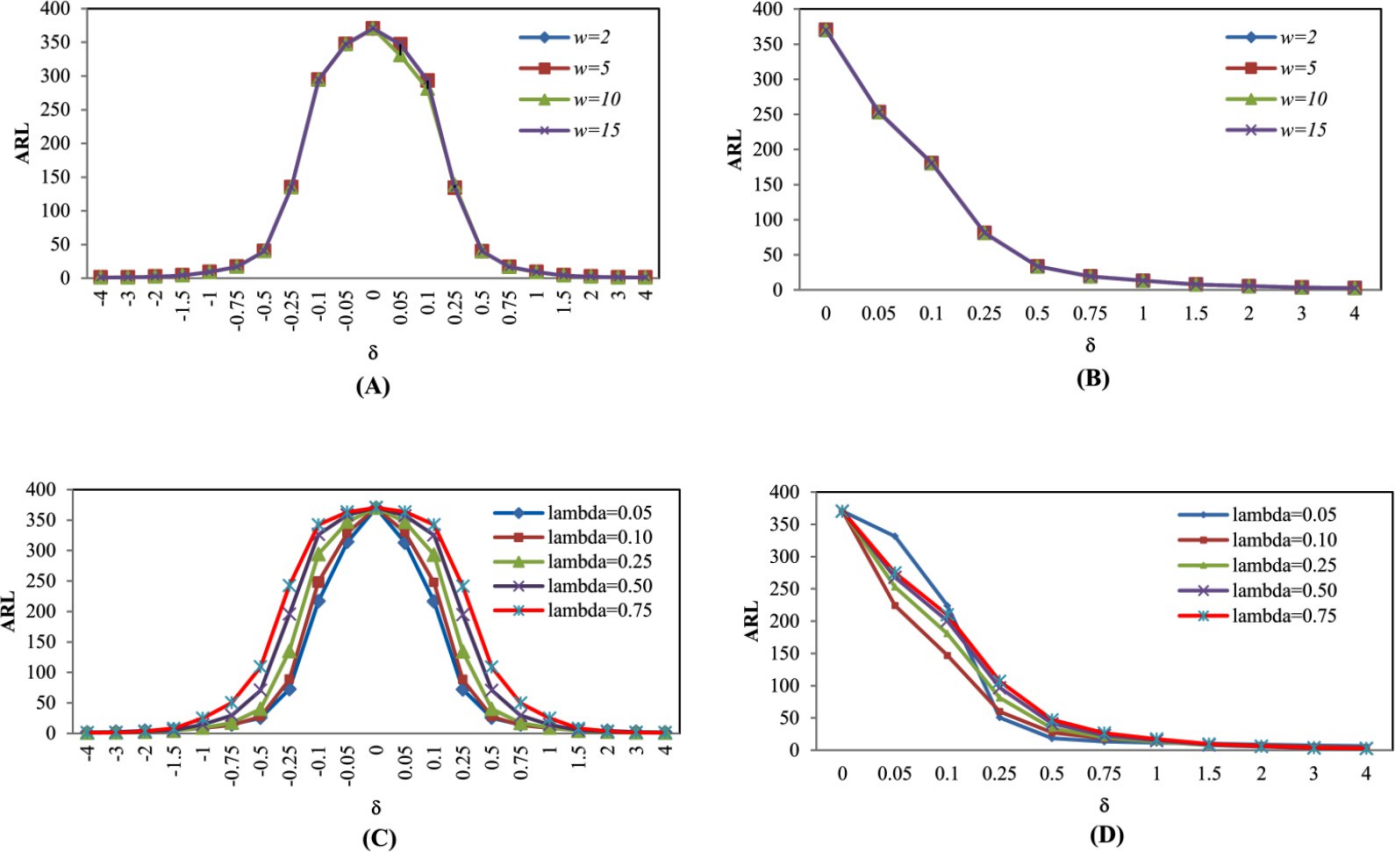
This is the Fig 2 ARL curves of EWMA-MA chart for Normal(0,1) and Exponential(1) distributions by varying *w* and *λ* (A) EWMA-MA chart for Normal(0,1) distribution by varying *w*. (B) EWMA-MA chart for Exponential(1) distribution by varying *w*. (C) EWMA-MA chart for Normal(0,1) distribution by varying *λ*. (D) EWMA-MA chart for Exponential(1) distribution by varying *λ*.

The simulation results are shown in Tables 1 and 2 varying *w* and Tables 3 and 4 varying *λ* when the distributed data is Normal(0,1) and Exponential(1). They are also illustrated in Fig 2, when span size *w* = 2, 5, 10 and 15. On the other hand, when changing *λ* = 0.05, 0.10, 0.25, 0.50 and 0.75, it can be seen that the obtained ARL_1_ is not different when changing *w*. Conversely, when changing parameter *λ*, the obtained ARL_1_ is different. From Table 5, when the data is distributed as Normal (0, 1) with *w* = 5 and *λ* = 0.05, 0.10, 0.25, 0.50 and 0.75, under moderate shifts *δ* = ±0.05, ±0.10, ±0.25 and ±0.50 where *λ* = 0.05 gives the minimum ARL_1_, while *δ* = ±0.75, ±1.00, where *λ* = 0.10 gives the minimum ARL_1_, while *δ* = ±1.50, ±2.00, where *λ* = 0.25 gives the minimum ARL_1_ and *δ* = ±3.00, ±4.00, when *λ* = 0.50, giving the least ARL_1_. Meanwhile, when the data is distributed as Exponential (1) as shown in Table 6, where *δ* = 0.05, 0.10, when *λ* = 0.10 gives the minimum ARL_1_, at *δ* = 0.25, 0.50, 0.75 and 1.00 with *λ* = 0.05 ARL_1_ is the least, *δ* = 1.50, 2.00 when *λ* = 0.25, ARL_1_ is the least and *δ* = 3.00, 4.00 when *λ* = 0.50, giving the least ARL_1_.

**Table 1:** ARL performance of EWMA-MA chart for Normal(0,1) distribution by varying *w* and given ARL_0_ = 370 and *λ* = 0.25.

Shift size (*δ*)	*w* = 2	*w* = 5	*w* = 10	*w* = 15
	*H*_*4*_ *=* 4.098	*H*_*4*_ *=* 6.480	*H*_*4*_ *=* 9.165	*H*_*4*_ *=* 11.225
-4.00	1.02±0.00	1.02±0.00	1.02±0.00	1.02±0.00
-3.00	1.27±0.00	1.27±0.00	1.27±0.00	1.27±0.00
-2.00	2.47±0.00	2.47±0.00	2.47±0.00	2.47±0.00
-1.50	4.18±0.01	4.19±0.01	4.19±0.01	4.19±0.01
-1.00	9.26±0.02	9.26±0.02	9.26±0.02	9.26±0.02
-0.75	17.00±0.03	17.00±0.03	17.01±0.03	17.01±0.03
-0.50	40.09±0.08	40.10±0.08	40.11±0.08	40.11±0.08
-0.25	134.90±0.29	134.98±0.29	135.05±0.29	135.06±0.29
-0.10	294.12±0.65	294.27±0.65	294.54±0.65	294.57±0.65
-0.05	346.89±0.77	347.12±0.77	347.43±0.77	347.50±0.77
0.00	370.10±0.82	370.32±0.82	370.67±0.83	370.75±0.83
0.05	346.67±0.77	346.89±0.77	330.66±0.67	347.23±0.77
0.10	292.87±0.65	293.07±0.65	280.90±0.64	293.31±0.65
0.25	134.16±0.29	134.23±0.29	137.97±0.30	134.32±0.29
0.50	39.98±0.08	39.99±0.08	40.48±0.22	40.01±0.08
0.75	16.92±0.03	16.92±0.03	16.67±0.19	16.93±0.03
1.00	9.21±0.01	9.21±0.01	9.11±0.15	9.21±0.01
1.50	4.18±0.01	4.18±0.01	4.25±0.06	4.18±0.01
2.00	2.48±0.00	2.48±0.00	2.51±0.03	2.48±0.00
3.00	1.27±0.00	1.27±0.00	1.29±0.01	1.27±0.00
4.00	1.02±0.00	1.02±0.00	1.02±0.00	1.02±0.00

after the mark (±) is standard deviation of ARL_._

**Table 2:** ARL performance of EWMA-MA chart for Exponential(1) distribution by varying *w* and given ARL_0_ = 370 and *λ* = 0.25.

Shift size (*δ*)	*w* = 2	*w* = 5	*w* = 10	*w* = 15
	*H*_*4*_ *=* 5.220	*H*_*4*_ *=* 8.251	*H*_*4*_ *=* 11.667	*H*_*4*_ *=* 14.289
0.00	370.84±0.82	370.30±0.82	370.09±0.82	370.08±0.82
0.05	253.56±0.56	253.18±0.56	253.02±0.55	253.02±0.55
0.10	180.97±0.39	180.74±0.39	180.62±0.39	180.61±0.39
0.25	81.31±0.17	81.21±0.17	81.18±0.17	81.18±0.17
0.50	33.78±0.07	33.76±0.07	33.75±0.07	33.75±0.07
0.75	19.43±0.04	19.41±0.04	19.41±0.04	19.41±0.04
1.00	13.18±0.02	13.18±0.02	13.17±0.02	13.17±0.02
1.50	7.86±0.01	7.86±0.01	7.86±0.01	7.86±0.01
2.00	5.60±0.01	5.60±0.01	5.60±0.01	5.60±0.01
3.00	3.60±0.01	3.60±0.01	3.60±0.01	3.60±0.01
4.00	2.72±0.00	2.72±0.00	2.72±0.00	2.72±0.00

after the mark (±) is standard deviation of ARL_._

**Table 3:** ARL performance of EWMA-MA-chart for Normal(0,1) distribution by varying *λ* and given ARL_0_ = 370 and *w* = 5.

Shift size (*δ*)	*λ* = 0.05	*λ* = 0.10	*λ* = 0.25	*λ* = 0.50	*λ* = 0.75
	*H*_*4*_ *=* 5.568	*H*_*4*_ *=* 6.041	*H*_*4*_ *=* 6.480	*H*_*4*_ *=* 6.658	*H*_*4*_ *=* 6.698
-4.00	1.57±0.00	1.15±0.00	1.02±0.00	1.01*±0.00	1.03±0.00
-3.00	2.35±0.00	1.76±0.00	1.27±0.00	1.21*±0.00	1.37±0.00
-2.00	3.98±0.00	3.18±0.00	2.47*±0.00	2.55±0.00	3.55±0.01
-1.50	5.76±0.00	4.81±0.00	4.19*±0.01	5.06±0.01	8.03±0.02
-1.00	9.76±0.01	8.75*±0.01	9.26±0.02	14.30±0.03	24.81±0.05
-0.75	14.35±0.02	13.77*±0.02	17.00±0.03	29.30±0.06	49.95±0.11
-0.50	25.49*±0.03	27.29±0.04	40.10±0.08	70.82±0.16	109.19±0.24
-0.25	72.45*±0.13	88.50±0.18	134.98±0.29	195.52±0.43	242.47±0.54
-0.10	216.78*±0.45	248.37±0.54	294.27±0.65	326.42±0.73	342.49±0.76
-0.05	314.05*±0.67	329.12±0.72	347.12±0.77	358.46±0.80	363.35±0.81
0.00	370.16±0.80	370.15±0.81	370.32±0.82	370.52±0.83	370.59±0.83
0.05	312.99*±0.67	329.58±0.72	346.89±0.77	357.14±0.80	363.63±0.81
0.10	216.22*±0.45	247.16±0.54	293.07±0.65	325.39±0.72	342.39±0.76
0.25	72.02*±0.13	88.04±0.18	134.23±0.29	194.95±0.43	241.31±0.54
0.50	25.41*±0.03	27.22±0.04	39.99±0.08	70.52±0.15	108.54±0.24
0.75	14.28±0.02	13.70*±0.02	16.92±0.03	29.06±0.06	49.57±0.11
1.00	9.72±0.01	8.72*±0.01	9.21±0.01	14.25±0.03	24.71±0.05
1.50	5.75±0.00	4.80±0.00	4.18*±0.01	5.06±0.01	8.02±0.02
2.00	3.98±0.00	3.18±0.00	2.48*±0.00	2.55±0.00	3.54±0.01
3.00	2.35±0.00	1.76±0.00	1.27±0.00	1.21*±0.00	1.38±0.00
4.00	1.57±0.00	1.15±0.00	1.02±0.00	1.01*±0.00	1.03±0.00

* is minimal ARL_1_ and after the mark (±) is standard deviation of ARL.

**Table 4:** ARL performance of EWMA-MA chart for Exponential(1) distribution by varying *λ* and given ARL_0_ = 370 and *w* = 5.

Shift size (*δ*)	*λ* = 0.05	*λ* = 0.10	*λ* = 0.25	*λ* = 0.50	*λ* = 0.75
	*H*_*4*_ *=* 6.648	*H*_*4*_ *=* 7.858	*H*_*4*_ *=* 8.251	*H*_*4*_ *=* 9.850	*H*_*4*_ *=* 10.700
0.00	370.40±0.91	370.04±0.98	370.30±0.82	370.41±0.82	370.35±0.83
0.05	331.53±0.76	224.22*±0.85	253.18±0.56	267.97±0.60	274.93±0.61
0.10	224.02±0.62	147.05*±0.59	180.74±0.39	200.54±0.45	210.00±0.47
0.25	50.69*±0.47	59.97±0.21	81.21±0.17	96.93±0.21	106.80±0.24
0.50	18.39*±0.11	27.20±0.07	33.76±0.07	41.13±0.09	47.24±0.10
0.75	13.26*±0.06	18.01±0.04	19.41±0.04	22.89±0.05	26.60±0.06
1.00	11.56*±0.04	13.77±0.03	13.18±0.02	14.91±0.03	17.31±0.04
1.50	10.00±0.03	9.68±0.02	7.86*±0.01	8.28±0.02	9.51±0.02
2.00	8.98±0.02	7.55±0.01	5.60*±0.01	5.62±0.01	6.38±0.01
3.00	7.48±0.01	5.33±0.01	3.60±0.01	3.45*±0.01	3.87±0.01
4.00	6.35±0.01	4.14±0.01	2.72±0.00	2.56*±0.00	2.85±0.01

* is minimal ARL_1_ and after the mark (±) is standard deviation of ARL.

**Table 5:** ARL performance of MA-EWMA chart for Normal(0,1) distribution by varying *w* and given ARL_0_ = 370 and *λ* = 0.25.

Shift size (*δ*)	*w* = 2	*w* = 5	*w* = 10	*w* = 15
	*H*_*3*_ *=* 7.894	*H*_*3*_ *=* 7.632	*H*_*3*_ *=* 7.267	*H*_*3*_ *=* 7.000
-4.00	0.03±0.00	0.00*±0.00	0.00*±0.00	0.00*±0.00
-3.00	0.28±0.00	0.05±0.00	0.02±0.00	0.01*±0.00
-2.00	1.88±0.01	0.47±0.00	0.18±0.00	0.11*±0.00
-1.50	5.33±0.01	1.51±0.01	0.56±0.00	0.34*±0.00
-1.00	18.89±0.05	5.83±0.02	2.24±0.00	1.19*±0.01
-0.75	40.20±0.10	13.52±0.04	5.16±0.00	2.72*±0.01
-0.50	93.60±0.22	37.18±0.11	14.70±0.00	7.61*±0.04
-0.25	222.58±0.52	124.59±0.35	58.53±0.00	31.70*±0.16
-0.10	328.55±0.76	243.70±0.68	144.44±0.00	89.13*±0.44
-0.05	350.98±0.81	277.22±0.77	176.69±0.00	114.76*±0.57
0.00	370.07±0.83	370.05±0.83	370.50±0.00	370.82±0.84
0.05	351.20±0.81	276.83±0.77	176.88±0.00	114.16*±0.57
0.10	328.10±0.76	242.74±0.68	144.20±0.00	88.63*±0.44
0.25	222.46±0.52	124.39±0.35	58.50±0.00	31.68*±0.16
0.50	93.23±0.22	37.20±0.11	14.64±0.00	7.60*±0.04
0.75	40.13±0.10	13.52±0.04	5.12±0.00	2.67*±0.01
1.00	18.94±0.05	5.87±0.02	2.21±0.00	1.18*±0.01
1.50	5.31±0.01	1.53±0.01	0.56±0.00	0.34*±0.00
2.00	1.86±0.01	0.47±0.00	0.18±0.00	0.11*±0.00
3.00	0.28±0.00	0.05±0.00	0.02±0.00	0.01*±0.00
4.00	0.03±0.00	0.00*±0.00	0.00*±0.00	0.00*±0.00

* is minimal ARL_1_ and after the mark (±)is standard deviation of ARL.

**Table 6:** ARL performance of MA-EWMA chart for Exponential(1) distribution by varying *w* and given ARL_0_ = 370 and *λ* = 0.25.

Shift size (*δ*)	*w* = 2	*w* = 5	*w* = 10	*w* = 15
	*H*_*3*_ *=* 5.4700	*H*_*3*_ *=* 4.4240	*H*_*3*_ *=* 3.7935	*H*_*3*_ *=* 3.4900
0.00	370.93±0.83	370.28±0.84	370.15±0.85	370.00±0.86
0.05	264.13±0.60	228.20±0.57	166.98±0.52	97.09*±0.43
0.10	198.03±0.45	160.43±0.40	110.95±0.35	63.38*±0.28
0.25	95.30±0.22	67.96±0.18	42.54±0.14	23.12*±0.11
0.50	39.69±0.09	25.41±0.07	14.85±0.05	8.01*±0.04
0.75	21.41±0.05	13.04±0.04	7.51±0.03	4.06*±0.02
1.00	13.45±0.03	8.03±0.02	4.63±0.02	2.54*±0.01
1.50	6.95±0.02	4.11±0.01	2.36±0.01	1.35*±0.01
2.00	4.42±0.01	2.59±0.01	1.50±0.01	0.89*±0.00
3.00	2.40±0.01	1.40±0.01	0.83±0.00	0.53*±0.00
4.00	1.60±0.00	0.93±0.00	0.57±0.00	0.38*±0.00

* is minimal ARL_1_ and after the mark (±) is standard deviation of ARL.

### Expansion of the MA-EWMA control chart

**Fig 3:**
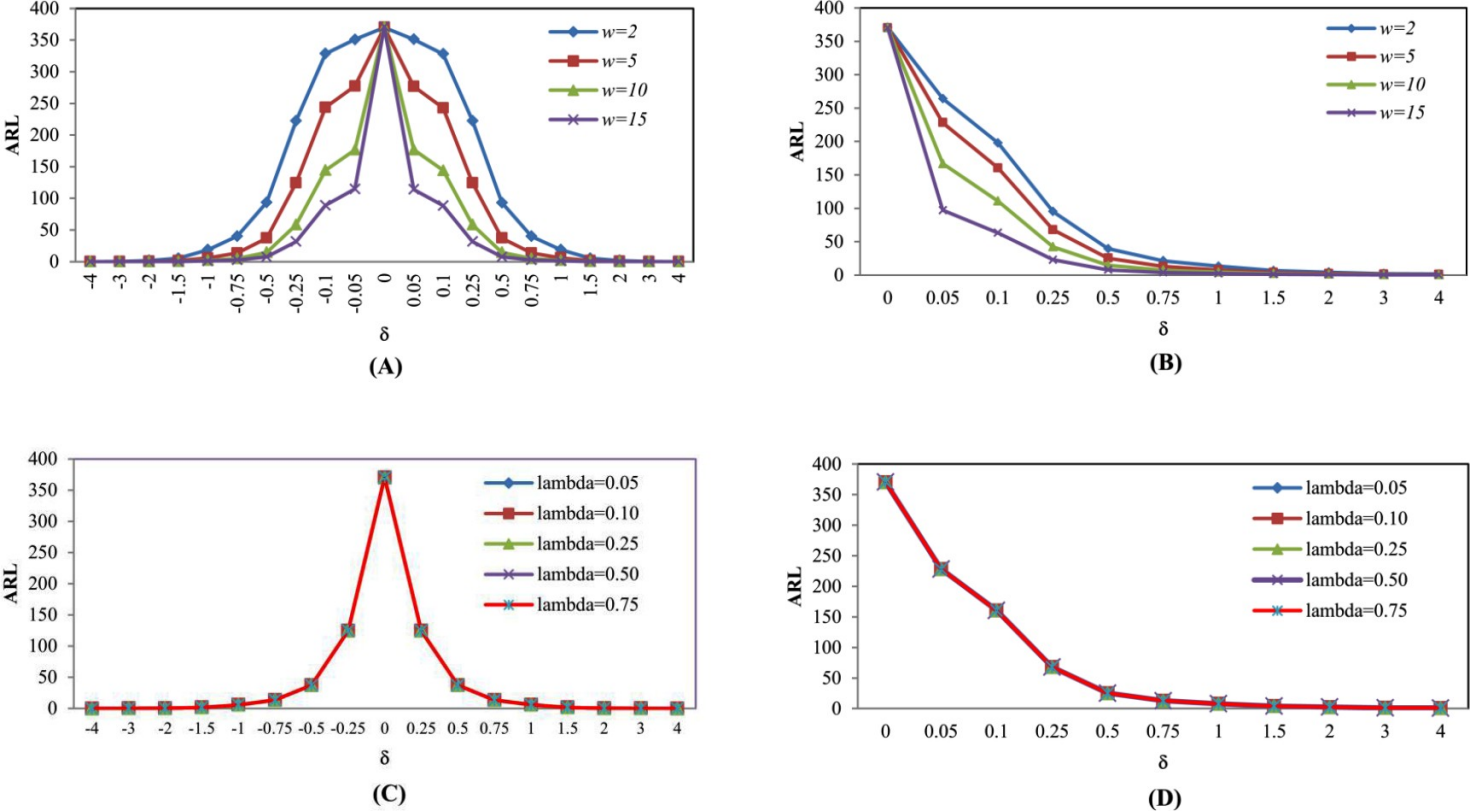
This is the Fig 3 ARL curves of MA-EWMA chart for Normal(0,1) and Exponential(1) distributions by varying *w* and *λ*. (A) MA-EWMA chart for Normal(0,1) distribution by varying *w*. (B) MA-EWMA chart for Exponential(1) distribution by varying *w*. (C) MA-EWMA chart for Normal(0,1) distribution by varying *λ*. (D) MA-EWMA chart for Exponential(1) distribution by varying *λ*.

From the MA-EWMA chart was presented by Taboran et al. [28], the researcher expanded on the work by changing *w* and *λ*, as shown in Tables 5–8. When the distributions are Normal(0,1) and Exponential(1), MA-EWMA will present a decreasing ARL_1_ as *w* increases. When changing *λ* = 0.05, 0.10, 0.25, 0.50 and 0.75, ARL_1_ is almost the same without much difference, as shown in Fig 3, it can be seen that the obtained ARL_1_ is less if parameter *w* is greater.

**Table 7:** ARL performance of MA-EWMA chart for Normal(0,1) distribution by varying *λ* and given ARL_0_ = 370 and *w* = 5.

Shift size (*δ*)	*λ* = 0.05	*λ* = 0.10	*λ* = 0.25	*λ* = 0.50	*λ* = 0.75
	*H*_*3*_ *=* 18.020	*H*_*3*_ *=* 12.575	*H*_*3*_ *=* 7.267	*H*_*3*_ *=* 4.997	*H*_*3*_ *=* 3.725
-4.00	0.00±0.00	0.00±0.00	0.00±0.00	0.00±0.00	0.00±0.00
-3.00	0.05±0.00	0.05±0.00	0.05±0.00	0.05±0.00	0.05±0.00
-2.00	0.47±0.00	0.47±0.00	0.47±0.00	0.47±0.00	0.47±0.00
-1.50	1.51±0.01	1.51±0.01	1.51±0.01	1.51±0.01	1.51±0.01
-1.00	5.83±0.02	5.83±0.02	5.83±0.02	5.83±0.02	5.83±0.02
-0.75	13.54±0.04	13.53±0.04	13.52±0.04	13.53±0.04	13.54±0.04
-0.50	37.25±0.11	37.20±0.11	37.18±0.11	37.21±0.11	37.24±0.11
-0.25	124.93±0.35	124.71±0.35	124.59±0.35	124.76±0.35	124.88±0.35
-0.10	243.70±0.68	243.70±0.68	243.70±0.68	243.70±0.68	243.70±0.68
-0.05	277.22±0.77	277.22±0.77	277.22±0.77	277.22±0.77	277.22±0.77
0.00	370.95±0.83	370.37±0.83	370.05±0.83	370.47±0.83	370.81±0.83
0.05	276.83±0.77	276.83±0.77	276.83±0.77	276.83±0.77	276.83±0.77
0.10	242.74±0.68	242.74±0.68	242.74±0.68	242.74±0.68	242.74±0.68
0.25	124.69±0.35	124.48±0.35	124.39±0.35	124.51±0.35	124.62±0.35
0.50	37.27±0.11	37.22±0.11	37.20±0.11	37.23±0.11	37.26±0.11
0.75	13.54±0.04	13.53±0.04	13.52±0.04	13.53±0.04	13.54±0.04
1.00	5.87±0.02	5.87±0.02	5.87±0.02	5.87±0.02	5.87±0.02
1.50	1.53±0.01	1.53±0.01	1.53±0.01	1.53±0.01	1.53±0.01
2.00	0.47±0.00	0.47±0.00	0.47±0.00	0.47±0.00	0.47±0.00
3.00	0.05±0.00	0.05±0.00	0.05±0.00	0.05±0.00	0.05±0.00
4.00	0.00±0.00	0.00±0.00	0.00±0.00	0.00±0.00	0.00±0.00

after the mark (±) is standard deviation of ARL

**Table 8:** ARL performance of MA-EWMA chart for Exponential(1) distribution by varying *λ* and given ARL_0_ = 370 and *w* = 5.

Shift size (*δ*)	*λ* = 0.05	*λ* = 0.10	*λ* = 0.25	*λ* = 0.50	*λ* = 0.75
	*H*_*3*_ *=* 4.6700	*H*_*3*_ *=* 4.6100	*H*_*3*_ *=* 4.4240	*H*_*3*_ *=* 4.0970	*H*_*3*_ *=* 3.7400
0.00	370.294±0.839	370.424±0.839	370.28±0.84	370.805±0.840	370.80±0.84
0.05	228.208±0.568	228.268±0.569	228.20±0.57	228.500±0.569	228.49±0.57
0.10	160.434±0.404	160.488±0.404	160.43±0.40	160.637±0.404	160.63±0.40
0.25	67.959±0.177	67.972±0.177	67.96±0.18	68.017±0.177	68.02±0.18
0.50	25.411±0.069	25.415±0.069	25.41±0.07	25.428±0.069	25.43±0.07
0.75	13.043±0.037	13.045±0.037	13.04±0.04	13.052±0.037	13.05±0.04
1.00	8.035±0.024	8.036±0.024	8.03±0.02	8.039±0.024	8.04±0.02
1.50	4.115±0.013	4.115±0.013	4.11±0.01	4.117±0.013	4.12±0.01
2.00	2.594±0.009	2.595±0.009	2.59±0.01	2.595±0.009	2.60±0.01
3.00	1.402±0.005	1.402±0.005	1.40±0.01	1.402±0.005	1.40±0.01
4.00	0.932±0.004	0.932±0.004	0.93±0.00	0.932±0.004	0.93±0.00

after the mark (±) is standard deviation of ARL

### Performance comparisons

From this research, the study was under the six distributions processes, which were symmetrical distributions: Normal(0,1), Laplace(0,1), Logistic(6,2), and Student *t*_10_ distributions, and asymmetric distributions: Exponential(1) and Gamma(4,1) distributions, that compared the efficiency in detecting the change of the control chart when the change was as shown in the following table.

**Fig 4:**
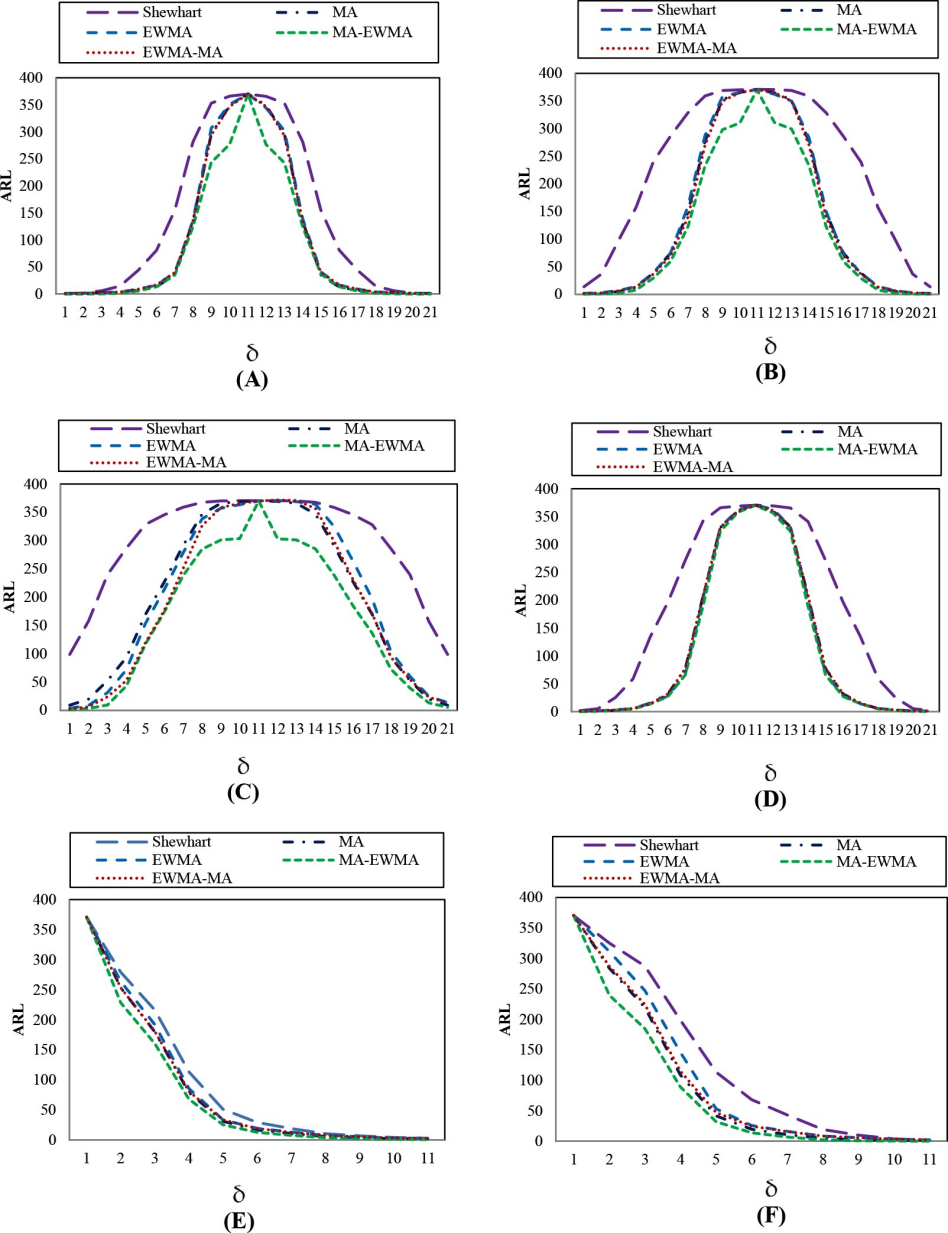
This is the Fig 4 Comparative ARL performance of Shewhart, MA, EWMA, MA-EWMA and EWMA-MA charts with w = 5, *λ* = 0.25 and ARL0 = 370, under the symmetrical distributions:(A) Normal(0,1), (B) Laplace(0,1), (C) Logistic(6,2), (D) Student t10, and asymmetric distributions: (E) Exponential(1), and (F) Gamma(4,1) distributions.

**Fig 5:**
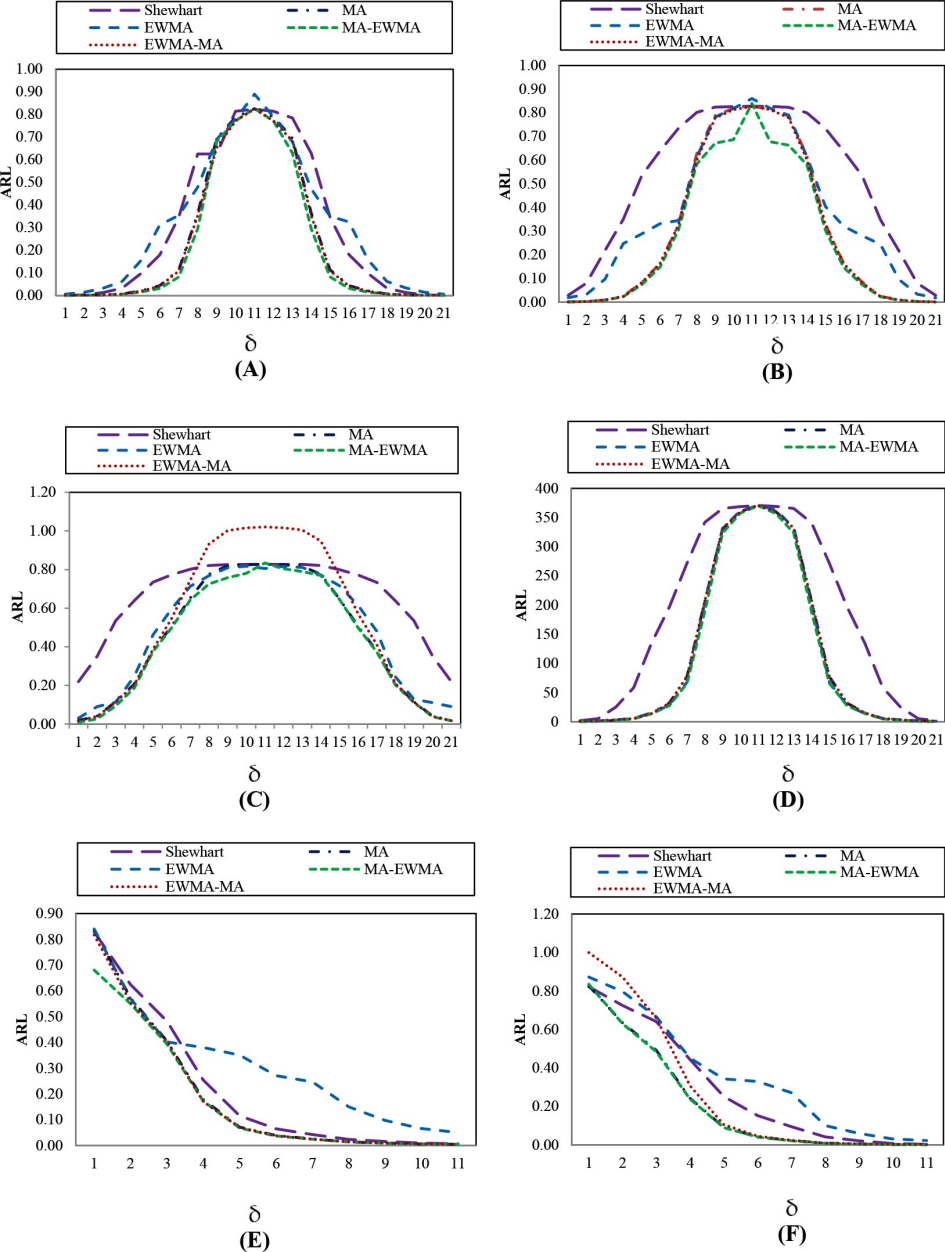
This is the Fig 5 Comparative SDRL performance of Shewhart, MA, EWMA, MA-EWMA and EWMA-MA charts with w = 5, *λ* = 0.25 and ARL0 = 370, under the symmetrical distributions:(A) Normal(0,1), (B) Laplace(0,1), (C) Logistic(6,2), (D) Student t10, and asymmetric distributions: (E) Exponential(1), and (F) Gamma(4,1) distributions.

**Fig 6:**
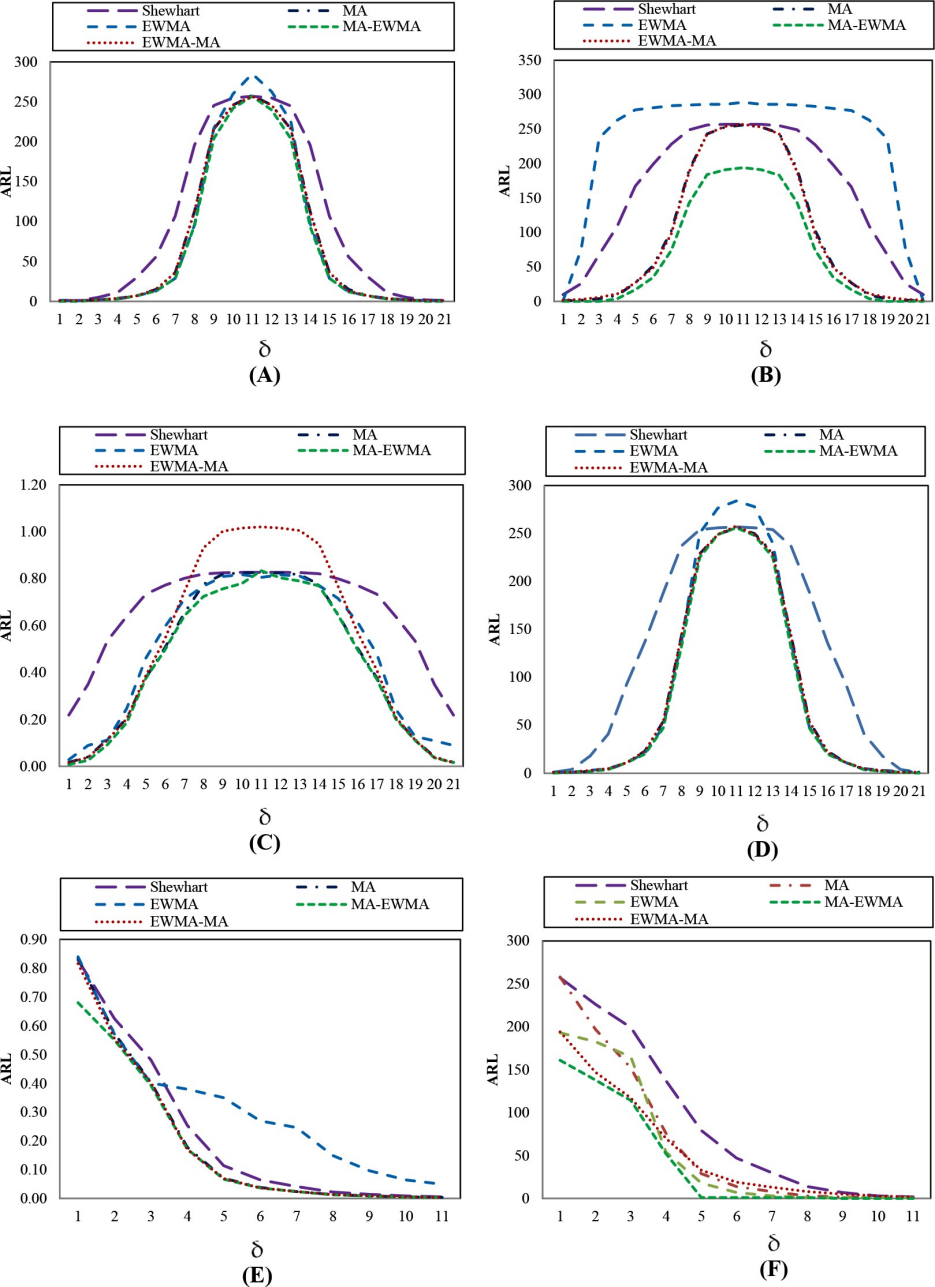
This is the Fig 6 Comparative MRL performance of Shewhart, MA, EWMA, MA-EWMA and EWMA-MA charts with w = 5, *λ* = 0.25 and ARL0 = 370, under the symmetrical distributions:(A) Normal(0,1), (B) Laplace(0,1), (C) Logistic(6,2), (D) Student t10, and asymmetric distributions: (E) Exponential(1), and (F) Gamma(4,1) distributions.

Tables 9–13 shows the ARL, SDRL, and MRL in observation simulated from different distributions, distributed data Normal(0,1), Laplace(0,1), Logistic(6,2), Student *t*_10_, Exponential(1), and Gamma(4,1), showed that *H*_4_ = 6.480, *H*_4_ = 7.455, *H*_4_ = 20.292, *H*_4_ = 7.640, *H*_4_ = 8.251 and *H*_4_ = 7.600 (where *w* = 5, *λ* = 0.25) of EWMA-MA chart, the ARL_1_, SDRL and MRL were lower than Shewhart, MA, and EWMA charts for all magnitudes of change. For comparison EWMA-MA with MA-EWMA charts, the performance of MA-EWMA control chart is superior to EWMA-MA control chart for all shifts, as shown in Figs 4–6.

**Table 9:** Performance comparison of the proposed charts with *w* = 5, *λ* = 0.25 and ARL_0_ = 370 under the Normal(0,1) distribution.

*δ*	Shewhart			MA			EWMA			MA-EWMA			EWMA-MA		
	*H = 3*.*000*			*H*_*1*_ *= 3*.*000*			*H*_*2*_ *= 2*.*927*			*H*_*3*_ *= 7*.*632*			*H*_*4*_ *= 6*.*480*		
	*ARL*	*SDRL*	*MRL*	*ARL*	*SDRL*	*MRL*	*ARL*	*SDRL*	*MRL*	*ARL*	*SDRL*	*MRL*	*ARL*	*SDRL*	*MRL*
-4.00	1.19	0.00	1.00	1.16	0.00	1.00	1.08	0.01	1.00	**0.00**	0.00	**0.00**	1.02	0.00	1.00
-3.00	2.00	0.00	1.00	1.62	0.00	1.00	1.38	0.01	1.00	**0.05**	0.00	**0.00**	1.27	0.00	1.00
-2.00	6.34	0.01	5.00	2.81	0.00	2.00	2.51	0.03	2.00	**0.47**	0.00	2.00	2.47	0.00	2.00
-1.50	14.98	0.03	11.00	3.84	0.01	3.00	4.19	0.06	4.00	**1.51**	0.01	3.00	4.19	0.01	4.00
-1.00	43.83	0.10	30.00	6.97	0.02	7.00	9.09	0.15	7.00	**5.83**	0.02	7.00	9.26	0.02	7.00
-0.75	81.05	0.18	56.00	13.15	0.04	15.00	16.72	0.31	13.00	**13.52**	**0.03**	13.00	17.00	0.04	15.00
-0.50	155.20	0.35	107.00	37.18	0.11	36.00	39.93	0.35	29.00	**35.51**	**0.08**	29.00	40.10	0.11	36.00
-0.25	281.36	0.63	196.00	134.51	0.36	113.00	136.26	0.48	96.00	**124.59**	**0.29**	**95.00**	134.98	0.35	113.00
-0.10	353.25	0.63	245.50	296.16	0.69	216.00	307.28	0.69	217.00	**243.70**	0.63	**204.00**	294.27	0.65	216.00
-0.05	366.04	0.81	255.00	348.91	0.79	246.00	351.09	0.78	260.00	**277.22**	0.77	**242.00**	347.11	0.77	246.00
0.00	370.00	0.82	257.00	370.40	0.83	258.00	370.00	0.89	285.00	370.05	0.82	257.00	370.32	0.82	257.00
0.05	365.82	0.81	255.00	348.91	0.79	246.00	344.66	0.79	263.00	**276.83**	0.77	**240.00**	346.89	0.77	246.00
0.10	353.03	0.79	245.00	296.16	0.69	216.00	302.15	0.68	226.00	**242.74**	**0.63**	**204.00**	293.07	0.68	216.00
0.25	281.60	0.63	196.00	134.51	0.36	113.00	138.84	0.47	98.00	**124.39**	**0.29**	**94.00**	134.23	0.35	112.00
0.50	155.23	0.35	107.00	35.51	0.11	36.00	40.76	0.35	29.00	**37.20**	**0.08**	29.00	39.99	0.11	36.00
0.75	81.23	0.18	56.00	13.15	0.04	15.00	16.70	0.33	12.00	**13.52**	**0.03**	12.00	16.92	0.04	13.00
1.00	43.92	0.10	31.00	6.97	0.02	7.00	9.11	0.16	7.00	**5.87**	**0.01**	7.00	9.21	0.02	7.00
1.50	14.99	0.03	11.00	3.84	0.01	3.00	4.18	0.06	4.00	**1.53**	0.01	3.00	4.18	0.01	4.00
2.00	6.33	0.01	5.00	2.81	0.00	2.00	2.54	0.03	2.00	**0.47**	0.00	2.00	2.48	0.00	2.00
3.00	2.00	0.00	2.00	1.62	0.00	1.00	1.39	0.01	1.00	**0.05**	0.00	0.00	1.27	0.00	1.00
4.00	1.19	0.00	1.00	1.16	0.00	1.00	1.08	0.01	1.00	**0.00**	0.00	**0.00**	1.02	0.00	1.00

The bold is minimal of ARL, SDRL and MRL

**Table 10:** Performance comparison of the proposed charts with *w* = 5, *λ* = 0.25 and ARL_0_ = 370 under the Student t_10_ distribution.

*δ*	Shewhart			MA			EWMA			MA-EWMA			EWMA-MA		
	*H = 3*.*958*			*H*_*1*_ *= 3*.*314*			*H*_*2*_ *= 3*.*465*			*H*_*3*_ *= 8*.*778*			*H*_*4*_ *= 7*.*640*		
	*ARL*	*SDRL*	*MRL*	*ARL*	*SDRL*	*MRL*	*ARL*	*SDRL*	*MRL*	*ARL*	*SDRL*	*MRL*	*ARL*	*SDRL*	*MRL*
-4.00	1.94	0.00	1.00	1.02	0.00	1.00	1.18	0.01	1.00	**0.27**	0.00	**0.00**	1.09	0.00	1.00
-3.00	5.56	0.01	4.00	1.24	0.00	1.00	1.67	0.02	1.00	**0.85**	0.00	1.00	1.61	0.00	1.00
-2.00	25.33	0.06	18.00	2.66	0.00	2.00	3.35	0.04	3.00	**2.51**	0.00	2.00	3.33	0.00	3.00
-1.50	58.62	0.13	41.00	5.32	0.01	4.00	5.87	0.09	5.00	**5.22**	0.01	4.00	5.86	0.01	5.00
-1.00	134.32	0.30	93.00	15.37	0.03	11.00	15.26	0.26	11.00	**13.91**	0.03	11.00	14.11	0.03	11.00
-0.75	196.28	0.44	137.00	31.99	0.07	23.00	28.01	0.36	21.00	**27.43**	0.07	**20.00**	31.89	0.07	23.00
-0.50	271.76	0.61	188.00	77.57	0.17	54.00	67.98	0.45	48.50	**66.05**	0.17	**47.00**	77.71	0.17	54.00
-0.25	341.86	0.76	237.00	206.56	0.46	143.00	203.98	0.54	137.00	**189.04**	0.47	**132.00**	207.36	0.47	143.00
-0.10	365.70	0.82	254.00	330.53	0.74	229.00	331.30	0.74	250.50	**323.68**	**0.63**	**225.00**	331.00	0.72	229.00
-0.05	368.83	0.82	256.00	360.35	0.81	249.00	361.75	0.81	276.50	**357.74**	**0.68**	249.00	359.78	0.79	249.00
0.00	370.51	0.83	257.00	370.47	0.83	256.00	370.64	0.82	284.00	370.07	0.83	256.00	370.80	0.82	258.00
0.05	369.06	0.82	256.00	360.03	0.81	250.00	363.35	0.81	278.00	**356.68**	**0.68**	**248.00**	360.57	0.79	249.00
0.10	365.53	0.82	254.00	331.75	0.74	230.00	331.80	0.75	240.00	**323.77**	**0.65**	**225.00**	331.88	0.72	229.00
0.25	340.93	0.76	237.00	206.97	0.46	144.00	201.83	0.53	137.00	**188.80**	**0.42**	**131.00**	207.90	0.47	144.00
0.50	271.83	0.61	189.00	77.60	0.17	54.00	68.06	0.45	48.00	**66.00**	**0.14**	**47.00**	77.62	0.17	54.00
0.75	196.57	0.44	136.00	31.96	0.07	23.00	28.09	0.36	21.00	**27.44**	**0.05**	**20.00**	32.04	0.07	22.00
1.00	134.33	0.30	93.00	15.39	0.03	11.00	14.18	0.26	11.00	**14.13**	**0.02**	11.00	15.35	0.03	11.00
1.50	58.82	0.13	41.00	5.31	0.01	4.00	5.90	0.08	5.00	**5.21**	0.01	4.00	5.83	0.01	5.00
2.00	25.36	0.06	18.00	2.65	0.00	2.00	3.39	0.04	3.00	**2.52**	0.00	2.00	3.33	0.00	3.00
3.00	5.54	0.01	4.00	1.24	0.00	1.00	1.73	0.02	2.00	**0.85**	0.00	1.00	1.60	0.00	1.00
4.00	1.94	0.00	1.00	1.02	0.00	1.00	1.18	0.01	1.00	**0.27**	0.00	**0.00**	1.09	0.00	1.00

The bold is minimal of ARL, SDRL and MRL.

**Table 11:** Performance comparison of the proposed charts with *w* = 5, *λ* = 0.25 and ARL_0_ = 370 under the Laplace(0,1)distribution.

*δ*	Shewhart			MA			EWMA			MA-EWMA			EWMA-MA		
	*H = 3*.*112*			*H*_*1*_ *= 3*.*396*			*H*_*2*_ *= 2*.*927*			*H*_*3*_ *= 8*.*242*			*H*_*4*_ *= 7*.*455*		
	*ARL*	*SDRL*	*MRL*	*ARL*	*SDRL*	*MRL*	*ARL*	*SDRL*	*MRL*	*ARL*	*SDRL*	*MRL*	*ARL*	*SDRL*	*MRL*
-4.00	13.57	0.03	10.00	1.20	0.00	1.00	1.789	0.02	1.00	**0.08**	0.00	**0.00**	1.67	0.00	2.00
-3.00	36.78	0.08	26.00	1.98	0.00	2.00	2.929	0.03	76.00	**0.34**	0.00	**0.00**	2.82	0.00	3.00
-2.00	98.16	0.22	68.00	5.37	0.01	4.00	6.876	0.10	237.00	**2.37**	0.01	**0.00**	6.59	0.01	6.00
-1.50	157.19	0.35	109.00	12.59	0.03	9.00	14.011	0.25	263.00	**7.94**	0.02	**4.00**	13.33	0.02	11.00
-1.00	239.88	0.53	167.00	38.69	0.08	27.00	38.940	0.29	278.00	**29.32**	**0.07**	**17.00**	36.68	0.08	27.00
-0.75	286.24	0.64	199.00	74.40	0.16	52.00	74.357	0.33	281.00	**59.36**	**0.15**	**35.00**	69.53	0.16	49.00
-0.50	328.52	0.73	228.00	147.66	0.33	102.00	159.050	0.35	284.00	**122.07**	**0.30**	**74.00**	139.76	0.33	98.00
-0.25	359.02	0.80	249.00	277.65	0.63	191.00	287.644	0.61	285.00	**232.58**	**0.58**	**143.00**	270.37	0.60	188.00
-0.10	368.91	0.82	256.00	352.11	0.79	243.00	357.635	0.78	286.00	**298.00**	**0.67**	**184.00**	349.75	0.78	243.00
-0.05	370.11	0.83	257.00	365.43	0.82	253.00	365.471	0.82	286.00	**310.33**	**0.69**	**191.00**	364.94	0.81	254.00
0.00	370.80	0.83	257.00	370.36	0.83	256.00	370.419	0.86	289.00	370.17	0.83	194.00	370.56	0.83	257.00
0.05	370.46	0.83	257.00	364.71	0.81	253.00	362.054	0.82	286.00	**310.87**	**0.68**	**191.00**	364.98	0.81	253.00
0.10	368.83	0.82	255.00	351.64	0.79	244.00	349.881	0.79	286.00	**299.15**	**0.66**	**183.00**	349.78	0.78	243.00
0.25	358.55	0.80	249.00	276.56	0.62	192.00	283.412	0.61	285.00	**233.04**	**0.58**	**143.00**	270.17	0.60	188.00
0.50	328.30	0.73	227.00	147.88	0.33	102.00	150.026	0.40	283.00	**121.98**	**0.30**	**74.00**	138.99	0.33	97.00
0.75	285.57	0.64	198.00	74.46	0.16	52.00	72.004	0.32	280.00	**59.19**	**0.15**	**35.00**	69.24	0.16	49.00
1.00	239.35	0.53	166.00	38.79	0.08	27.00	38.234	0.28	277.00	**29.23**	**0.07**	**17.00**	36.77	0.08	27.00
1.50	156.62	0.35	109.00	12.52	0.03	9.00	13.951	0.24	263.00	**7.92**	0.02	**4.00**	13.38	0.02	11.00
2.00	97.90	0.22	68.00	5.35	0.01	4.00	6.694	0.10	236.00	**2.37**	0.01	**0.00**	6.60	0.01	6.00
3.00	36.71	0.08	26.00	1.98	0.00	2.00	2.877	0.03	77.00	**0.34**	0.00	**0.00**	2.83	0.00	3.00
4.00	13.52	0.03	10.00	1.20	0.00	1.00	1.767	0.02	1.00	**0.08**	0.00	**0.00**	1.67	0.00	2.00

The bold is minimal of ARL, SDRL and MRL.

**Table 12:** Performance comparison of the proposed charts with *w* = 5, *λ* = 0.25 and ARL_0_ = 370 under the Logistic(4,1) distribution.

*δ*	Shewhart			MA			EWMA			MA-EWMA			EWMA-MA		
	*H = 9*.*338*			*H*_*1*_ *= 7*.*661*			*H*_*2*_ *= 3*.*298*			*H*_*3*_ *= 18*.*340*			*H*_*4*_ *= 20*.*292*		
	*ARL*	*SDRL*	*MRL*	*ARL*	*SDRL*	*MRL*	*ARL*	*SDRL*	*MRL*	*ARL*	*SDRL*	*MRL*	*ARL*	*SDRL*	*MRL*
-4.00	98.96	0.22	69.00	9.14	0.02	7.00	3.562	0.03	1.00	**1.16**	**0.01**	1.00	2.44	0.02	1.00
-3.00	157.86	0.35	110.00	19.77	0.04	14.00	8.793	0.09	1.00	**3.12**	**0.03**	1.00	6.24	0.04	7.00
-2.00	240.31	0.54	167.00	52.79	0.12	37.00	31.522	0.11	4.00	**9.62**	**0.09**	**2.00**	24.10	0.11	22.00
-1.50	286.51	0.64	199.00	93.56	0.21	65.00	71.995	0.25	18.00	**43.03**	**0.19**	**10.00**	52.77	0.20	42.00
-1.00	328.68	0.73	228.00	169.55	0.38	118.00	153.758	0.46	60.00	**117.08**	**0.38**	**46.00**	119.58	0.39	80.00
-0.75	345.78	0.77	240.00	226.65	0.51	157.00	213.528	0.60	97.00	**173.75**	**0.50**	**80.00**	176.50	0.55	110.00
-0.50	358.95	0.80	249.00	291.18	0.65	201.00	278.331	0.71	151.00	**238.00**	**0.64**	**127.00**	250.70	0.75	142.00
-0.25	367.79	0.82	255.00	346.92	0.78	240.00	338.663	0.77	210.00	**285.01**	**0.73**	**171.00**	326.45	0.93	180.00
-0.10	370.03	0.83	257.00	366.16	0.82	253.00	357.462	0.81	238.00	**301.07**	**0.76**	**181.00**	358.56	1.00	204.00
-0.05	370.61	0.83	257.00	369.05	0.83	255.00	363.750	0.82	246.00	**303.7**	**0.78**	**183.00**	365.60	1.02	211.00
0.00	370.69	0.83	257.00	370.19	0.83	256.00	370.123	0.81	258.00	370.31	0.83	256.00	370.39	1.02	216.00
0.05	370.64	0.83	257.00	369.43	0.82	256.00	370.985	0.82	257.00	**303.39**	**0.80**	**183.00**	371.75	1.02	219.00
0.10	370.37	0.83	256.00	366.20	0.82	254.00	368.944	0.81	255.00	**301.27**	**0.79**	**181.00**	371.20	1.01	221.00
0.25	367.76	0.82	255.00	346.99	0.78	241.00	364.445	0.77	249.00	**285.52**	**0.77**	**171.00**	356.74	0.95	218.00
0.50	358.49	0.80	248.00	290.34	0.65	202.00	324.302	0.72	213.00	**238.49**	**0.65**	**142.00**	298.33	0.77	188.00
0.75	345.84	0.77	240.00	226.53	0.51	157.00	262.914	0.62	169.50	**184.02**	**0.50**	**109.00**	230.08	0.57	150.00
1.00	328.39	0.73	227.00	170.06	0.38	118.00	197.353	0.48	129.00	**136.77**	**0.37**	**81.00**	170.97	0.41	113.00
1.50	285.82	0.64	199.00	93.46	0.21	65.00	103.969	0.24	72.00	**72.69**	**0.20**	**42.00**	93.72	0.21	65.00
2.00	239.74	0.53	166.00	52.75	0.11	37.00	60.003	0.13	44.00	**39.56**	**0.11**	**22.00**	54.31	0.11	40.00
3.00	157.24	0.35	109.00	19.75	0.04	14.00	24.910	0.11	19.00	**13.13**	**0.04**	**7.00**	23.13	0.04	18.00
4.00	98.74	0.22	69.00	9.12	0.02	7.00	13.579	0.09	11.00	**5.13**	**0.02**	**1.00**	13.01	0.02	11.00

The bold is minimal of ARL, SDRL and MRL.

**Table 13:** Performance comparison of the proposed charts with *w* = 5, *λ* = 0.25 and ARL_0_ = 370 under the Exponential(1), and Gamma(4,1) distributions.

*δ*		Exponential(1)					Gamma(4,1)				
		Shewhart	MA	EWMA	MA-EWMA	EWMA-MA	Shewhart	MA	EWMA	MA-EWMA	EWMA-MA
		*H*	*H*_*1*_	*H*_*2*_	*H*_*3*_	*H*_*4*_	*H*	*H*_*1*_	*H*_*2*_	*H*_*3*_	*H*_*4*_
		(4.915)	(3.339)	(3.747)	(4.424)	(8.251)	(3.894)	(3.023)	(3.549)	(2.005)	(7.600)
0.00	ARL	370.11	370.82	370.378	370.28	370.30	370.18	370.52	370.04	370.29	370.60
	SDRL	0.83	0.83	0.84	0.68	0.82	0.82	0.83	0.87	0.84	1.00
	MRL	256.00	257.00	278.00	223.00	258.00	257.0	258.00	193.0	161.0	194.00
0.05	ARL	279.28	253.40	263.70	**228.20**	253.18	325.07	283.92	311.13	**239.33**	286.90
	SDRL	0.62	0.57	0.57	0.57	0.56	0.72	0.63	0.80	0.63	0.87
	MRL	193.00	175.00	191.00	**149.00**	178.00	226.0	197.00	182.5	**138.0**	147.00
0.10	ARL	216.01	180.02	192.00	**160.43**	180.74	287.04	219.56	247.13	**184.63**	224.43
	SDRL	0.48	0.40	0.40	**0.39**	0.40	0.64	0.49	0.67	0.49	0.66
	MRL	150.00	124.00	139.50	**104.00**	127.00	199.0	152.00	165.0	**114.0**	117.00
0.25	ARL	113.40	78.99	85.583	**67.96**	81.21	198.65	108.91	145.89	**89.16**	114.93
	SDRL	0.25	0.18	0.38	0.17	0.17	0.44	0.24	0.45	0.24	0.31
	MRL	78.00	54.00	60.00	**42.00**	58.00	137.0	76.00	54.00	**52.0**	70.00
0.50	ARL	51.52	31.40	33.726	**25.41**	33.76	113.45	41.48	53.20	**31.97**	47.17
	SDRL	0.11	0.07	0.35	0.07	0.07	0.25	0.09	0.34	0.09	0.10
	MRL	36.00	22.00	25.00	**15.00**	25.00	79.0	29.00	18.00	**1.00**	33.00
0.75	ARL	29.33	17.01	19.550	**13.04**	19.41	68.21	19.52	25.79	**13.92**	24.76
	SDRL	0.06	0.04	0.27	0.04	0.04	0.15	0.04	0.33	0.04	0.05
	MRL	20.00	12.00	15.00	**7.00**	15.00	47.0	14.00	7.00	**1.00**	19.00
1.00	ARL	19.25	10.95	13.544	**8.03**	13.18	43.06	10.75	16.17	**6.99**	15.65
	SDRL	0.04	0.02	0.25	0.02	0.02	0.09	0.02	0.27	0.02	0.02
	MRL	13.00	8.00	10.00	**4.00**	10.00	30.00	8.00	3.00	**1.00**	13.00
1.50	ARL	10.63	6.09	7.985	**4.11**	7.86	19.44	4.53	8.50	**2.31**	8.49
	SDRL	0.02	0.01	0.15	0.01	0.01	0.04	0.01	0.10	0.01	0.01
	MRL	8.00	4.00	6.00	**1.00**	6.00	14.00	3.00	1.00	1.00	8.00
2.00	ARL	7.18	4.15	5.616	**2.59**	5.60	10.10	2.53	5.87	**0.93**	5.74
	SDRL	0.01	0.01	0.10	0.01	0.01	0.02	0.00	0.06	0.00	0.01
	MRL	5.00	3.00	5.00	**1.00**	5.00	7.00	2.00	1.00	**0.00**	5.00
3.00	ARL	4.38	2.59	3.644	**1.40**	3.60	3.81	1.32	3.49	**0.20**	3.41
	SDRL	0.01	0.01	0.06	0.01	0.01	0.01	0.00	0.03	0.00	0.00
	MRL	3.00	2.00	3.00	**0.00**	3.00	3.00	1.00	1.00	**0.00**	3.00
4.00	ARL	3.26	1.97	2.763	**0.93**	2.72	2.06	1.06	2.46	**0.04**	2.37
	SDRL	0.01	0.00	0.05	0.00	0.00	0.00	0.00	0.02	0.00	0.00
	MRL	2.00	1.00	2.00	**0.00**	2.00	2.00	1.00	1.00	**0.00**	2.00

The bold is minimal of ARL, SDRL and MRL.

## Practical applications

In this case study, the real data of the flow rate of Nile river between 1871–1930 [32] and real GDP growth (%) in the Lebanese economy data from International Monetary Fund (IMF) between 1970–2003 [33] can be found in S1 Table, based on the time-series data used in this study, therefore, the basic alternate is that the time series is stationary (or trend-stationary). In consequence, the concept and theory relating to the classical time series to test the stationary of data. This study applied Unit Root Test in the stationary test by using the Augmented Dickey Fuller Test (ADF) [34]. The result shows that the studied data do not have a unit root and are stationary. According to the statistical assumptions to construct variables quality parametric control charts [35], they were found that the studied data agree to the assumptions which contains normal distribution and independence. We have considered two applications: the data for flow in the Nile River and data of the real GDP growth (%) in the Lebanese economy.

### Application I: the Nile river flow rate between 1871–1930

**Fig 7:**
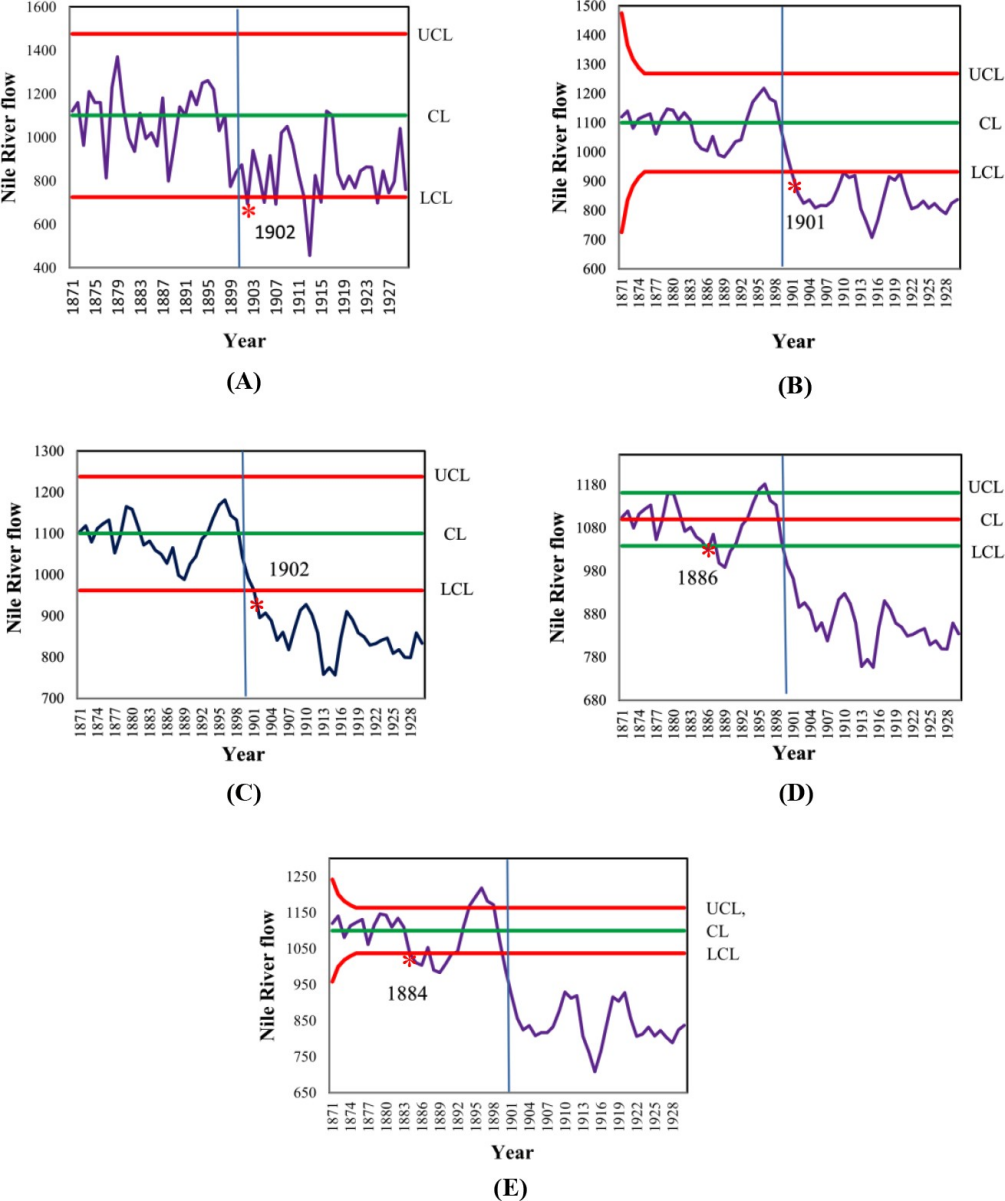
**This is the Fig 7 The performance comparison of detecting of a change in the Nile river flow rate (1871–1930) between Shewhart, MA, EWMA, MA-EWMA and EWMA-MA control charts,** (A) Shewhart chart, (B) MA chart, (C) EWMA chart, (D) EWMA-MA chart and (E) MA-EWMA chart.

From the data of the Nile river flow rate between 1871–1930 with normal distribution when the process had not changed and the mean of process at 1,100 m^3^/seconds had not changed. In 1900, the process changed, so the mean decreased to 850 m^3^/second with standard deviation of 125m^3^/seconds. The data generated the Shewhart, MA, EWMA, MA-EWMA and EWMA-MA charts as Eqs (3), (7), (9) and (11), as shown in Fig 7. It can be concluded that the MA-EWMA chart was the quickest to detect the change of flow rate recorded in the Nile River between 1871 and 1930 which was able to detect change of flow rate in 1884, while Shewhart chart was able to detect change of flow rate in 1902, MA chart was able to detect change of flow rate in 1901, EWMA chart was able to detect change of flow rate in 1902, EWMA-MA chart was able to detect change of flow rate in 1886 as shown on Fig 7. Therefore, MA-EWMA chart performs the best which is quickest detection a change early.

### Application II: the data of the real GDP growth (%) in the Lebanese economy

**Fig 8:**
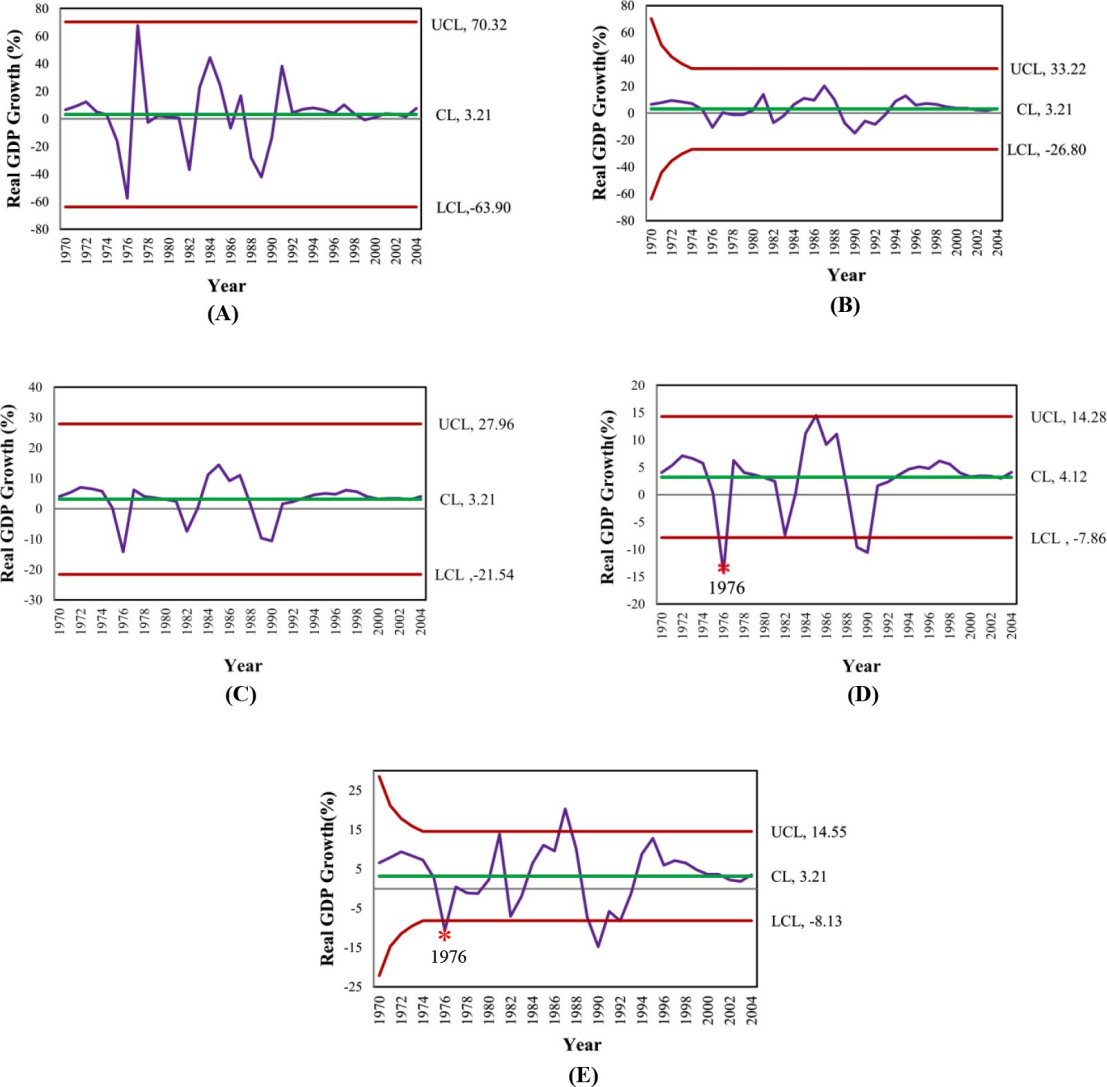
**This is the Fig 8 The performance comparison of detecting of a change for the real GDP growth (%) in the Lebanese economy covering the period 1970–2003 between Shewhart, MA, EWMA, MA-EWMA and EWMA-MA control charts**, (A) Shewhart chart, (B) MA chart, (C) EWMA chart, (D) EWMA-MA chart and (E) MA-EWMA chart.

For this application, we have selected real GDP growth (%) in the Lebanese economy data between 1970–2003 with normal distribution. The performance in detecting a mean of the real GDP growth (%) of Shewhart, MA, EWMA, MA-EWMA and EWMA-MA control charts are demonstrated in term of graphical results as Fig 8A), 8B), 8C), 8D) and 8E), respectively. For this case study, the performance of MA-EWMA and EWMA-MA control chart can detect a mean change at 1976 whereas the Shewhart, MA and EWMA control charts could not detect any change.

## Conclusions, discussions and recommendations

In this research, a new control chart is proposed, called the EWMA-MA chart to detect changes in the mean of the process in cases where the change process is under control ARL_0_ = 370. From research, the results showed that the proposed chart has better detection efficiency than the Shewhart, MA, and EWMA charts for all levels of changes when studying under asymmetric distributions with right skew. When studying under a process with symmetric distributions, there are some distributions that give minimally different results. Overall, however, the proposed chart exhibited better performance compared to the Shewhart, MA, and EWMA charts.

When comparing the proposed chart with the MA-EWMA chart under a process with asymmetric and symmetric distributions, it is found that the MA-EWMA chart has better efficiency in detecting changes of parameters than the EWMA-MA chart for all levels of changes. However, from the results of comparison, it is found that the MA-EWMA and EWMA-MA charts have ARL_1_ depending on the parameters of statistics of such control chart. That is MA-EWMA chart, when varying span size *w*, ARL_1_ will be different. However, ARL_1_ will not be different if *λ* has been changed. On the contrary, ARL_1_ will be different for the EWMA-MA chart when changing *λ*. However, if span size *w* is changed, ARL_1_ will not be different. From applying the proposed control chart to the data for flow in the Nile River and the real GDP growth (%) in the Lebanese economy, it is found to be in accordance with the research results.

In addition, the researchers compared the performance of MA-EWMA are compared with DEWMA mean [36], GWMA-CUSUM [37], and Progressive Mean [38] control charts. The results found that the MA-EWMA chart with that of the DEWMA mean chart by simulating the observations to have normal distribution (*μ* = 0, *σ* = 1) when the in-control ARL is 200. It was found that the DEWMA mean is more sensitive than the MA-EWMA chart when *δ*≤1.00 but if the shifts size of *δ* >1.00, the two control charts will share the similar efficiency in detecting the change. Besides, the comparison between the MA-EWMA control chart, the GWMA-CUSUM control chart at the in-control ARL is 500, and the Progressive Mean (PM) control chart at the in-control ARL is 370, found that the MA-EWMA chart performed better than the GWMA-CUSUM and PM charts for all magnitudes of change, except for at *δ* = 0.25, the GWMA-CUSUM and PM charts performed better than the MA-EWMA chart. However, it depends on the different parameters of each control chart due to the fact that each charts, for example, if setting *w* of the parameter in a large number, the ARL_1_ of the MA-EWMA control chart will be lower.

The results of the real GDP growth (%) in the Lebanese economy were consistent with Harvie et al. [39] study that the timing of major structural breaks in the Lebanese economy by applying the Zivot and Andrews (ZA) procedure, using annual time series data spanning the years from 1970 through 2003. the timing of the structural breaks for the real GDP growth (%) occurred in the years 1987, which are also the years when the country experienced a significant degree of macroeconomic and political instability. These findings therefore confirm the proposed charting method can used to for monitoring, controlling and can be applied to other fields such as health care, epidemiology, environmental sciences, etc. However, the adapted data must be in accordance with the charting statistic and the control limits depend on this assumption and as such the properties of parametric control chart.

In future studies, the scope of the study may be extended in terms of sample size, the method used in determining the ARL, and the process under control in other cases including the application to real data with other distributions, such as asymmetric distributions.

## Supporting information

S1 Table This is the S1 Table The real data of the flow rate of Nile river between 1871–1930 and real GDP growth (%) in the Lebanese economy data between 1970–2003. (DOCX)
