# Supplementary material for: Exponentially weighted moving average—Moving average charts for monitoring the process mean
Source: PLoS One. 2020 Feb 14;15(2):e0228208. doi: 10.1371/journal.pone.0228208 (PMC7021313; doi:10.1371/journal.pone.0228208)
Supplement: S1 Table — (DOCX) [file pone.0228208.s001.docx]

**Application I:** The data of the Nile river flow rate between 1871-1930.

| **Index** | **Year** | **Flow rate** | **Index** | **Year** | **Flow rate** |
| --- | --- | --- | --- | --- | --- |
| 1 | 1871 | 1120 | 31 | 1901 | 874 |
| 2 | 1872 | 1160 | 32 | 1902 | 694 |
| 3 | 1873 | 963 | 33 | 1903 | 940 |
| 4 | 1874 | 1210 | 34 | 1904 | 833 |
| 5 | 1875 | 1160 | 35 | 1905 | 701 |
| 6 | 1876 | 1160 | 36 | 1906 | 916 |
| 7 | 1877 | 813 | 37 | 1907 | 692 |
| 8 | 1878 | 1230 | 38 | 1908 | 1020 |
| 9 | 1879 | 1370 | 39 | 1909 | 1050 |
| 10 | 1880 | 1140 | 40 | 1910 | 969 |
| 11 | 1881 | 995 | 41 | 1911 | 831 |
| 12 | 1882 | 935 | 42 | 1912 | 726 |
| 13 | 1883 | 1110 | 43 | 1913 | 456 |
| 14 | 1884 | 994 | 44 | 1914 | 824 |
| 15 | 1885 | 1020 | 45 | 1915 | 702 |
| 16 | 1886 | 960 | 46 | 1916 | 1120 |
| 17 | 1887 | 1180 | 47 | 1917 | 1100 |
| 18 | 1888 | 799 | 48 | 1918 | 832 |
| 19 | 1889 | 958 | 49 | 1919 | 764 |
| 20 | 1890 | 1140 | 50 | 1920 | 821 |
| 21 | 1891 | 1100 | 51 | 1921 | 768 |
| 22 | 1892 | 1210 | 52 | 1922 | 845 |
| 23 | 1893 | 1150 | 53 | 1923 | 864 |
| 24 | 1894 | 1250 | 54 | 1924 | 862 |
| 25 | 1895 | 1260 | 55 | 1925 | 698 |
| 26 | 1896 | 1220 | 56 | 1926 | 845 |
| 27 | 1897 | 1030 | 57 | 1927 | 744 |
| 28 | 1898 | 1100 | 58 | 1928 | 796 |
| 29 | 1899 | 774 | 59 | 1929 | 1040 |
| 30 | 1900 | 840 | 60 | 1930 | 759 |

(Source:Wu Y. Inference for change-point and post-change means after a CUSUM test. Lecture Notes in Statistics 180. New York: Springer; 2005.)

**Application II:** The Lebanese economy data between 1970-2003.

| **Index** | **Year** | **Real GDP Growth (%)** | **Index** | **Year** | **Real GDP Growth (%)** |
| --- | --- | --- | --- | --- | --- |
| 1 | 1970 | 6.59 | 18 | 1987 | 16.72 |
| 2 | 1971 | 9.23 | 19 | 1988 | -28.21 |
| 3 | 1972 | 12.35 | 20 | 1989 | -42.18 |
| 4 | 1973 | 5.27 | 21 | 1990 | -13.43 |
| 5 | 1974 | 3.11 | 22 | 1991 | 38.27 |
| 6 | 1975 | -16.14 | 23 | 1992 | 4.50 |
| 7 | 1976 | -57.59 | 24 | 1993 | 7.05 |
| 8 | 1977 | 67.71 | 25 | 1994 | 8.00 |
| 9 | 1978 | -2.58 | 26 | 1995 | 6.50 |
| 10 | 1979 | 2.38 | 27 | 1996 | 4.00 |
| 11 | 1980 | 1.48 | 28 | 1997 | 10.20 |
| 12 | 1981 | 0.54 | 29 | 1998 | 3.90 |
| 13 | 1982 | -36.79 | 30 | 1999 | -0.80 |
| 14 | 1983 | 22.71 | 31 | 2000 | 1.10 |
| 15 | 1984 | 44.48 | 32 | 2001 | 3.90 |
| 16 | 1985 | 24.30 | 33 | 2002 | 3.40 |
| 17 | 1986 | -6.76 | 34 | 2003 | 1.70 |

(Source: International Monetary Fund. International Financial Statistics Yearbooks. Washington DC: Publication Services; 2004.)
